# Proteomic-Based Biosignatures in Breast Cancer Classification and Prediction of Therapeutic Response

**DOI:** 10.1155/2011/896476

**Published:** 2011-10-24

**Authors:** Jianbo He, Stephen A. Whelan, Ming Lu, Dejun Shen, Debra U. Chung, Romaine E. Saxton, Kym F. Faull, Julian P. Whitelegge, Helena R. Chang

**Affiliations:** ^1^Gonda/UCLA Breast Cancer Research Laboratory, David Geffen School of Medicine, University of California at Los Angeles, Los Angeles, CA 90095, USA; ^2^Department of Surgery, David Geffen School of Medicine, University of California at Los Angeles, Los Angeles, CA 90095, USA; ^3^Cardiovascular Proteomics Center, Center for Biomedical Mass Spectrometry, Boston University School of Medicine, Boston, MA 02118, USA; ^4^Department of Medicine, David Geffen School of Medicine, University of California at Los Angeles, Los Angeles, CA 90095, USA; ^5^Department of Pathology, Beth Israel Deaconess Medical Center, Harvard Medical School, Boston, MA 02215, USA; ^6^Pasarow Mass Spectrometry Laboratory, Semel Institute and Department of Psychiatry and Biobehavioral Science, David Geffen School of Medicine, University of California at Los Angeles, Los Angeles, CA 90095, USA; ^7^Revlon/UCLA Breast Center, David Geffen School of Medicine at UCLA, 200 UCLA Medical Plaza, B265, Los Angeles, CA 90095, USA

## Abstract

Protein-based markers that classify tumor subtypes and predict therapeutic response would be clinically useful in guiding patient treatment. We investigated the LC-MS/MS-identified protein biosignatures in 39 baseline breast cancer specimens including 28 HER2-positive and 11 triple-negative (TNBC) tumors. Twenty proteins were found to correctly classify all HER2 positive and 7 of the 11 TNBC tumors. Among them, galectin-3-binding protein and ALDH1A1 were found preferentially elevated in TNBC, whereas CK19, transferrin, transketolase, and thymosin *β*4 and *β*10 were elevated in HER2-positive cancers. In addition, several proteins such as enolase, vimentin, peroxiredoxin 5, Hsp 70, periostin precursor, RhoA, cathepsin D preproprotein, and annexin 1 were found to be associated with the tumor responses to treatment within each subtype. The MS-based proteomic findings appear promising in guiding tumor classification and predicting response. When sufficiently validated, some of these candidate protein markers could have great potential in improving breast cancer treatment.

## 1. Introduction

Chemotherapy has long been used to treat all types of cancer. Although survival benefits from adjuvant systemic chemotherapy in breast cancer have been thoroughly documented [1], success is not uniform with many still dying after the initial chemotherapy. The unpredictable tumor response to chemotherapy in any given patient and the significant toxicity manifested in all demand a better strategy for delivering cancer therapy. 

In selective subtypes of breast cancer, therapies targeting specific signal transduction and/or metabolic pathways have been successful. For example, Herceptin for HER2/neu positive breast cancer [2, 3] and poly(ADP ribose) polymerase (PARP) inhibitors for triple-negative breast cancer with defective DNA-repair [4, 5] are among the recent successes of targeted therapy. The success of target therapy has led to an explosion of interest in developing tailored systemic therapy. 

Breast cancer is a heterogeneous disease molecularly, histologically, and clinically. Clinical outcomes from the same treatment vary widely even among patients with tumors of identical stage and histology. Breast cancers developed from an accumulation of genetic alterations may partially explain the differences observed including tumor responses to anticancer agents [6]. Recently gene expression analysis has identified five subtypes of breast cancer which overlaps with clinical tumor classification according to the expression of three biomarkers, estrogen receptor (ER), progesterone receptor (PR), and epidermal growth factor receptor 2 (HER2). Clinically these three markers are prognostically and therapeutically important in guiding treatment selection [7–10]; however, they do not fully reflect the complexity and heterogeneity of breast cancer and do not always predict the outcome of the treatment. For example, Herceptin as a single agent or in combination with chemotherapy has been shown to reduce recurrent disease and to save lives in patients with HER2-positive breast cancer, yet a significant number of HER2 overexpression tumors do not respond to the treatment [11]. Additional molecular targets are expected to improve tailored treatment in the future. 

Proteomics has been employed in recent years to identify new disease-related biomarkers for cancer diagnosis and implementation of tailored treatment [12–15]. The tumor proteomes representing a global protein expression of cancer may provide new insights into the molecules that govern the dynamic cellular activities of tumor cells. Therefore, we choose to study breast tumor protein signatures in breast cancer classification and in predicting tumor response to treatment. 

Previously we used SELDI mass spectrometry to profile tumor response to neoadjuvant treatment and found that significant m/z profile differences existed between cancers of nonresponders (tumor regression rate ≤25%) and others (tumor regression rate >25%) [16]. In this current study we have applied the LC-MS/MS technology to study the breast cancer proteomes in human tissues and identify unique proteins that may have the potential to separate two subtypes of breast cancer (TNBC versus HER2+) and to predict drug responses within each subtype.

## 2. Materials and Methods

### 2.1. Collection of Breast Tumor Tissues and Classification of Response

Breast tumors were collected, processed, and banked as previously described [16]. This study was approved by the UCLA institutional review board (IRB). Tumors from 39 consented patients with locally advanced breast cancer were collected from a neoadjuvant clinical trial [17]. Eleven were triple-negative breast tumors (TNBC, ER-/PR-/HER2-), and 28 were HER2-positive tumors (HER2+). The tumor specimens were uniformly collected according to a standard operating procedure established in our laboratory. Baseline tumor specimens were obtained by either core needle biopsy or surgical biopsy before starting the neoadjuvant Taxotere/Carboplatin/±Herceptin treatment (TC ± H). Evaluation of tumor response to the treatment was measured both by pathologic examination of surgically removed tissue and by clinical assessment including physical examination and/or imaging studies. The pathological response of the tumor was reported as either pathologically complete response (pCR) or having residual tumor. Because a baseline tumor size by pathologic evaluation was not possible in patients receiving neoadjuvant treatment, the clinically or imaging-measured tumor size prior to chemotherapy was used as the baseline tumor size. Pathological assessment after chemotherapy including tumor size, lymph node staging, and tumor biomarkers was performed on the specimen obtained from the definitive breast cancer surgery [16]. The tumor regression rate (TRR) was used to evaluate tumor response induced by neoadjuvant therapy, and it was calculated as follows: (baseline tumor size − residual tumor size)/baseline tumor size × 100%, where the baseline tumor size was measured clinically, and the postchemotherapy residual invasive tumor size was measured pathologically. The tumor response was categorized into three groups: responders (TRR > 75%, R), intermediate responders (25% < TRR ≤ 75%, IR), and nonresponders (TRR ≤ 25%, NR).

### 2.2. Protein Extraction and Abundant Protein Depletion

Protein extraction from tumors and depletion of abundant proteins from tumor lysates were performed as previously described [16]. Briefly, frozen tumors were homogenized in liquid nitrogen and suspended in 1% Triton X-100. The samples were refrozen at −80°C and thawed on ice twice. Following centrifugation (10,000 g, 10 min, 4°C), the supernatants were subjected to albumin and immunoglobulin depletion using an albumin and IgG removal kit (Amersham) as well as hemoglobin depletion using Ni-NTA magnetic agarose beads (Qiagen). Protein concentrations of each preparation were determined by the BioRad protein assays. 

Because the blood proteins in the breast cancer tissue can cause significant ion suppression of lower abundance cancer-related proteins/peptides which may mask ion signals of less abundant peptides with similar M/Z ratios and retention times. In addition, the over presentation of serum proteins in the specimen may lower the amount of the cancer-related proteins available for LC-MS/MS analysis [18]. As a result, selected abundant serum proteins were depleted from the tissue extracts. Our preliminary test has shown more than 95% albumin, IgG and hemoglobin were removed by the described method, and more meaningful proteins have been detected. 

### 2.3. Trypsin Digestion

The dried protein samples were dissolved in 6 M guanidine HCl, reduced with DTT (5 mM–15 mM), and alkylated using 10 mM iodoacetamide. Samples were then diluted with NH_4_HCO_3_ to lower guanidine HCl concentration (1 M), mixed with trypsin (1 : 50 w/w ratio, sequencing grade, Promega) containing 50 mM ammonium bicarbonate, and incubated at 37°C overnight. Samples were desalted by C18 Microspin columns (The Nest Group), and the eluates were dried in a vacuum centrifuge.

### 2.4. LC-MS/MS Analysis

Each digested and dried sample was prepared for LC-MS/MS analysis as previously reported [19]. Briefly the samples were redissolved in Buffer A (H_2_O/acetonitrile/formic acid, 98.9/1/0.1, typically at 0.7 *μ*g protein/uL), and aliquots were injected (5 *μ*L) onto an in-house-prepared C18 trap. The retained materials were placed onto a reverse phase column (New Objective C18, 15 cm, 75 *μ*M diameter, 5 *μ*m particle size equilibrated in Buffer A) and eluted (300 nL/min, Eksigent Nanolc-2D) with an increasing concentration of Buffer B (acetonitrile/water/formic acid, 98.9/1/0.1; min/%B: 0/5, 10/10, 112/40, 130/60, 135/90, 140/90). Eluted peptides were analyzed by MS and data-dependent MS/MS (collision-induced dissociation) using online data-dependent tandem mass spectrometry (LTQ Orbitrap, Thermo Fisher Scientific) in which the seven most abundant precursor ions were selected for MS/MS. Before testing the experimental specimens, the reproducibility of LC-MS/MS analysis was confirmed by examining the triplicates of two different tissue samples, and similar proteins were identified from the triplicates of each sample with more than 90% overlapping. 

### 2.5. Database Searching and Analysis

BioWorks software (version 3.3.1, Thermo Fisher Sceintific), based on the SEQUEST algorithm (SRF v.5, Thermo Fisher Scientific), was used to search the mass spectra against a human trypsin indexed database (human.fasta.hdr database, Version 12.2, 227246 entries) as described by Whelan et al. [19]. SEQUEST was searched with a fragment ion mass tolerance of 1.00 Da and a parent ion tolerance of 50 PPM. The search tolerated up to two missed trypsin cleavages with variable modifications for carboxyamidomethylation (57.02146 Da) and methionine oxidation (15.99492 Da). Scaffold (version 3.0.3, Proteome Software, Inc.) was used to validate MS/MS-based peptide and protein identifications. Peptide identifications were accepted if they could be established at greater than 95.0% probability as specified by the Peptide Prophet Algorithm [20]. Protein identifications were accepted if they could be established with a greater than 99.0% probability and contained at least 2 identified peptides. Protein probabilities were assigned by the Protein Prophet Algorithm [21]. 

From the resulting MS/MS protein identifications, a list of proteins was generated for each sample. A list of semi-quantitative protein abundances in the different samples was developed using the normalized spectrum counts of the identified tryptic peptides from each protein, as compiled by the Scaffold program. The protein lists and their relative abundances were then compared to find differentially expressed proteins between two groups. 

### 2.6. Statistic Analysis

The data files exported from Scaffold were further processed as Excel files. The top 60% (180) abundant proteins of the 315 identified proteins were further selected for hierarchical clustering and supervised classification studies. Those proteins with at least a 2-fold difference in mean spectral counts between any two groups were selected for analysis in the web-based Gene Expression Profile Analysis Suite (GEPAS, version 4.0, http://www.gepas.org). Five different classification algorithms were tested to select candidate markers in the GEPAS software: Support Vector Machines (SVM), K-Nearest Neighbor Clustering (KNN), Diagonal Linear Discriminant Analysis (DLDA), Prediction Analysis with Microarrays (PAM), and Self-Organizing Map (SOM).

### 2.7. Immunohistochemistry Staining

A small portion of each of the baseline tumors was embedded in OCT and stored at −80°C. Endogenous peroxidase activity was quenched with 0.6% hydrogen peroxide in methanol for 10 minutes, and endogenous biotin was eliminated by Biotin Blocking System (DAKO, x0590). After blocking with 1 : 5 diluted normal goat serum or fetal bovine serum, slides were incubated for 1 hour with primary antibody (CK19, mouse IgG, ready to use, DAKO; galectin-3-binding protein, Goat IgG, 1 : 200 dilution, R&D) and 30 minutes with biotinylated secondary antibody (biotinylated anti-mouse Ig, 1 : 800 dilution, DAKO; biotinylated anti-goat Ig, 1 : 200 dilution, Vector Labs). Antigen-antibody complexes were then detected by the StreptABComplex/HRP method (DAKO) using diaminobenzidine as a chromogenic substrate (DAKO). Immunostained slides were lightly counterstained with hematoxylin. For negative controls, primary antibodies were replaced by mouse IgG or goat IgG. 

## 3. Results

### 3.1. Patient Characteristics

The reported thirty-nine baseline tumor specimens included 28 HER2-positive breast cancers and 11 TNBC with HER2 status determined by fluorescence in situ hybridization (FISH) assay. Fifteen of the 28 HER2+ patients were randomized to receive TC, and the remaining 13 received TC and Herceptin (TCH) before surgery. All eleven patients with TNBC received neoadjuvant TC. Following the neoadjuvant treatment, 28 patients with HER2+ tumors showed 12 responders (R), including 7 with pathological complete response (pCR) and 5 with a tumor regression rate >75%, 12 intermediate responders (IR), and 4 nonresponders (NR). In the TNBC group, there were 7 responders including 6 pCR and 1 with tumor regression rate >75%, 3 IR, and 1 NR. The clinical characteristics and pathologic features of the 11 TNBC and 28 HER2+ cases are summarized in Tables 1(a) and 1(b).

### 3.2. Protein Comparison between HER2+ and TNBC Groups

Proteins identified by MS/MS from the 39 tumors showed that 48 proteins were only found in HER2+ tumors, 24 were only seen in TNBC, and 243 proteins were shared by both, but the quantity of the shared proteins differed widely in the two tumor types. In this study, we focused the analysis on the top 60% abundant proteins (180/315) detected in the 39 tumors. 

The 20 most abundant shared proteins by both subtypes of cancer were summarized in Table 2. Among them apolipoprotein A-I and D, enolase 1, tumor rejection antigen (gp96) 1, transgelin 2, cofilin 1, profilin, heat shock proteins 70, and annexins 5 were found to be present in significant quantity in both types of breast cancer. Some of these shared proteins found in sufficient amount may be useful for breast cancer detection. 

**Figure 1:**
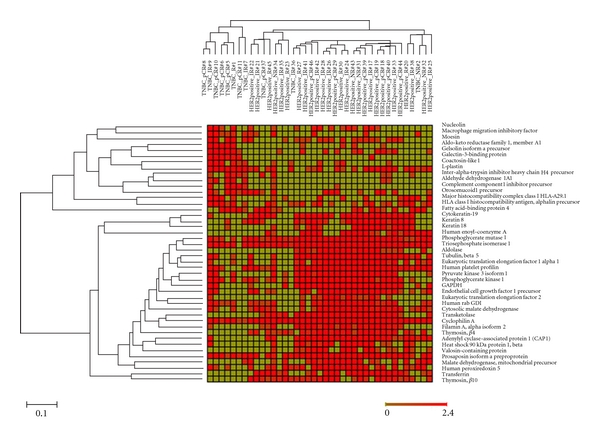
Heat map displaying the expression of 44 proteins in 28 HER2-positive and 11 TNBC tumors. Classification of 39 breast cancer cases into 2 groups based on tumor subtypes (HER-positive tumors and TNBC tumors) by the hierarchical clustering using GEPAS software. Each column represents a case as labeled on top, the short labeling cases are “TNBC” with sample ID, and long labeling cases are “HER2-positive” with sample ID. Each row represents a protein ID as indicated at the right. 44 proteins were expressed by ≥2-fold differences and detected in ≥50% of the cases in either group.

Of the 180 top abundant proteins observed in the 39 breast cancer specimens, 61 were found to have a ≥2-fold difference of spectrum counts between the two subtypes of breast cancer (HER2+ versus TNBC). Because some of these proteins were not detected in every sample, we further refined the list of differential proteins by selecting only those detected in ≥50% of the cases in either group. The selected 44 differentially expressed proteins were tested by hierarchical clustering to classify HER2+ breast cancer versus TNBC. These differentially expressed proteins correctly classified all 28 HER2+ tumors and 8 of the 11 TNBC by unweighted pair-group method using arithmetic average (UPGMA) (Figure 1).

**Figure 2:**
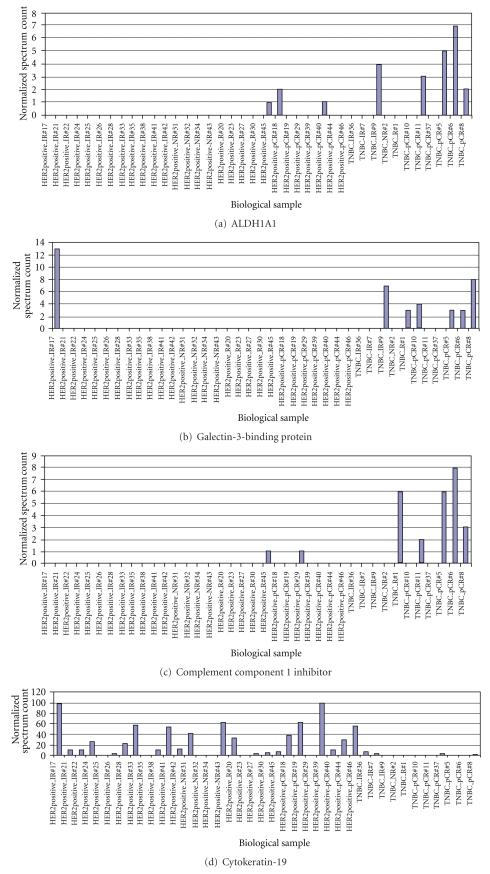

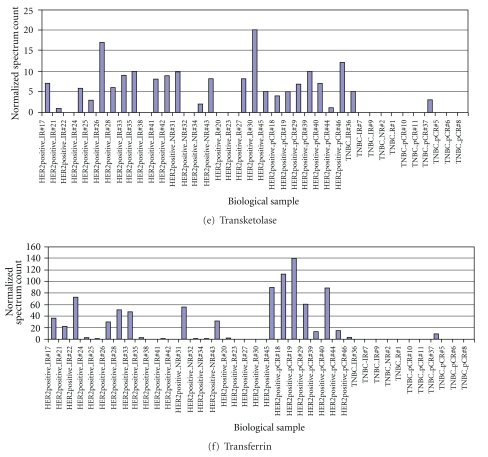
Representative proteins differentially expressed by HER2+ and TNBC tumors. (a)–(c): proteins preferentially expressed in TNBC. (d)-(e): proteins preferentially expressed in HER2+ tumors.

Self-validation of selected proteins in tumor classification was tested using a supervised classification. The 44 differentially expressed proteins were used to build a model separating subtypes of the tumors by Prophet, a web interface from the Gene Expression Profile Analysis Suite. Error rates were calculated as the number of misclassified tumors divided by total tumor cases tested. The error rates using various numbers of proteins by different models (SVM, KNN, DLDA, PAM, and SOM) were estimated by leaving-one-out tests (see File A in Supplementary Material available online at doi:10.1155/2011/896476). SVM had the lowest error rate (10%, 4/39) with 90% accuracy in tumor classification. The top 20 protein candidates (Table 3) selected by SVM model successfully classified all 28 HER2+ tumors and 7 of the 11 TNBC. Among the 20 differentially expressed proteins, G3BP, ALDH1A1, and complement component 1 inhibitor overexpression were found to be associated with TNBC subtype, whereas overexpression of CK19, transferrin, transketolase, and thymosin *β*4 and *β*10 were associated with HER2+ tumors (Figure 2).

### 3.3. Proteins Correlated with Different Tumor Response to Neoadjuvant Treatment among HER2+ Tumors

Of the 28 HER2+ tumors, there were 12 R (including 7 with pCR), 12 IR, and 4 NR. We compared proteomic differences between the two groups with extreme tumor response (pCR and NR) and found that 48 of the 180 proteins had an expressional difference ≥2-fold between 7 pCR versus 4 NR tumors. Self-validation of these potential marker proteins by five supervised classification methods suggested that the KNN had the lowest error rate (9%, 1/11) in predicting tumor response (Files B and C). By using KNN = 1 method, 100% (4/4) NR and 85.7% (6/7) pCR were correctly grouped by 20 selected proteins (Table 4). Of the 20 proteins, overexpressions of enolase1, vimentin, and L-plastin in HER2-positive tumors were associated with pCR, whereas high level of heat shock proteins 70 (Hsp70) and peroxiredoxin 5 (Prx V) were found only in the NR cases. 

### 3.4. Proteins Predicting TNBC Tumor Response to Neoadjuvant Treatment

Among the 11 TNBC cases, there were 7 R (including 6 pCR), 3 IR, and 1 NR. Due to the small sample size, the proteins of responders' tumor (R) were compared to all the remaining tumors with less response (IR + NR). Sixty-three of 180 proteins had a ≥2-fold mean differences between the two groups of TNBC with different response to the same treatment. Self-validation of these proteins by five supervised classification methods was used to compare the accuracy in predicting a tumor response. Using DLDA method, 6 of 7 tumors in the R group and 3 of 4 tumors in IR/NR group were correctly classified by the 30 selected proteins (error rate 18%) (Files D and E). Of these 30 proteins, the increased heat shock 70 kDa protein 8, periostin, Ras homolog gene family member A (RhoA), actinin alpha 4, cathepsin D preproprotein, annexin 1, and several other proteins were associated with drug resistance in TNBC (Table 5).

### 3.5. Evaluation of CK19 and G3BP Expression in TNBC and HER2+ Frozen Tumors

**Figure 3:**
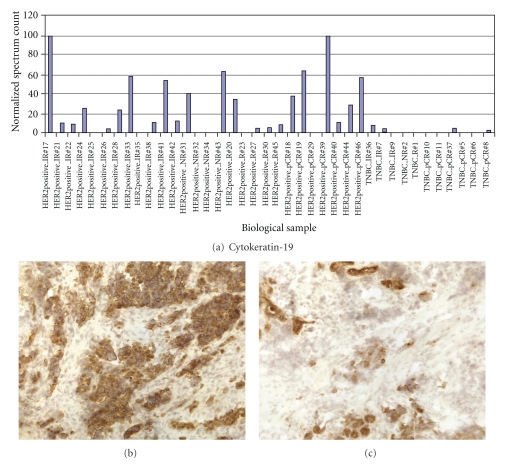
CK19 expressions detected by LC-MS/MS and immunohistochemical (IHC) staining. Elevated CK19 expressions found in HER+ tumor group by LC-MS/MS and confirmed by IHC in most of the frozen HER2+ tumors. (a) Normalized spectrum count of CK19 detected in 39 breast cancer tissues. (b) Immunohistochemical staining of CK19 in a HER2+ frozen tumor (power 200x). (c) Immunohistochemical staining of CK19 in a TNBC frozen tumor (power 200x).

**Figure 4:**
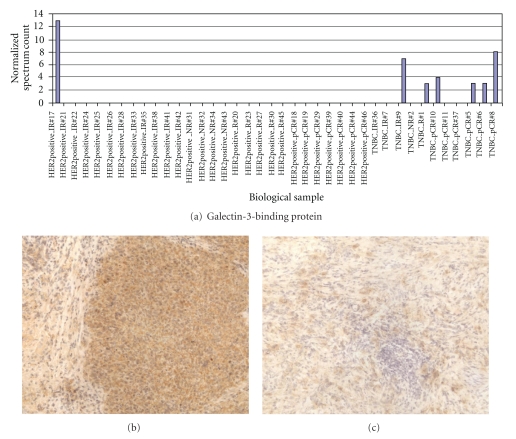
G3BP expressions detected by LC-MS/MS and immunohistochemical (IHC) staining. Elevated G3BP expressions found in TNBC group by LC-MS/MS and confirmed by IHC in most of the frozen TNBC tumors. (a) Normalized spectrum count of G3BP detected from 39 breast cancer tissues. (b) Immunohistochemical staining of G3BP in a TNBC frozen tumor (power 200x). (c) Immunohistochemical staining of G3BP in a HER2+ frozen tumor (power 200x).

CK19 and G3BP protein expressions were tested in breast cancer tumors by immunohistochemistry. The CK19 and G3BP staining showed cytoplasmic/membrane staining pattern in breast cancer cells. The overexpression of CK19 was found in HER2+ breast tumors (Figure 3) while the expression of G3BP was found to be upregulated in most TNBC (Figure 4). The concordance findings between the mass spectrometry analysis and immunohistochemical staining of the same tumor suggested that high-throughput mass spectrometry may be used as a screening tool to discover disease-related biomarkers. 

## 4. Discussion

In this discovery study, the MS-detected proteomic differences between two subtypes of breast cancer (HER2+ versus TNBC tumors) were explored, and proteomic prediction of tumor response to neoadjuvant chemotherapy was investigated. LC-MS/MS data sets of proteins from the 39 tumors analyzed allowed us to identify several candidate proteins that could classify tumor subtypes and predict tumor response to neoadjuvant chemotherapy.

Two clinical subtypes of breast cancer, HER2-positive and triple-negative breast cancers, defined by immunohistochemical staining and fluorescence in situ hybridization of three biomarkers of breast cancer have also been confirmed by gene analysis as two distinctive types of breast cancer. In this study, we reported that proteomic analysis could also separate the two subtypes by the unique biosignature associated with each type of breast cancer (Table 3). We also reported the potential of proteomic analysis in classifying drug-resistant TNBC and HER2+ breast cancer (Tables 4 and 5). 

Through an extensive literature review, some of the identified proteins have reported roles that are relevant to cancer biology and treatment. In TNBC tumors, we observed that the levels of G3BP, ALDH1A1, and complement component 1 inhibitor protein were preferentially elevated. All of them have been reported to have important biological properties in cancer progression. G3BP, also known as 90-kDa Mac-2-binding protein, is a member of the beta-galactoside-binding protein family and has a role in modulating cell-cell and cell-matrix interactions. It has been shown that G3BP is overexpressed in a variety of cancer cells such as colon, gastric, and breast cancer, and its overexpression appears to correlate with tumor progression, and metastasis [22–26]. Our report is the first to describe G3BP overexpression in human TNBC by both mass spectrometry analysis and immunohistochemical staining method. 

One protein correlated with triple-negative breast cancer meriting a discussion is ALDH1A1, a detoxifying enzyme responsible for oxidizing intracellular aldehydes. This process is important in early differentiation of stem cells through conversion of retinol to retinoic acid [27]. ALDH1 is considered to be a breast cancer stem cell marker and also a predictor for poor prognosis [27]. Because breast cancer stem cells have been implicated in radiation and chemotherapy resistance, as well as increasing the potential for metastasis, our finding of ALDH1A1 in TNBC may explain the more frequent relapse in TNBC patients. Previously, we have observed an overexpression of ALDH1 in TNBC when compared with hormone-receptor-positive and HER2-negative breast cancer [28]. In this paper, we also found a preferential overexpression of ALDH1 in TNBC over HER2-positive tumors. The unique overexpression of ALDH1 in TNBC tumors may point out an important population as the origin of some TNBC whereby notch signaling-dependent stem cell targets may be leveraged for target therapy development [29]. 

A different set of proteins was found preferentially elevated in HER2+ tumors. This list included CK19, transferrin, transketolase, and thymosin *β*4 and *β*10, and the biological significance of some of them will be discussed. 

Cytokeratins are known to be important in cellular motility, signaling, and division. While CK8/CK18 were similarly detected in both HER2+ and TNBC tumors, elevated CK19 was more commonly found in HER2+ tumors. Our observation coincides with the finding reported by Schulz et al. using a combination of 2D-DIGE/mass spectrometry and western blot [30]. Both our current and previous papers suggest that CK19 is low in TNBC when compared with either HER2-positive or HER2-negative but hormone-receptor-positive breast cancer [31]. Although the biological significance and the mechanism of reduced CK19 in TNBC are not clear, it could be related to the frequent recurrence and poor overall survival rate seen in TNBC patients [32]. 

Transferrin, another protein associated with HER2-positive cancer, is essential for cell growth and iron-dependent metabolic activities including DNA synthesis, electron transport, and mitogenic signaling pathways [33]. The elevation of transferrin receptor (CD71) was reported to be a marker of poor outcome [33]. Vyhlidal et al. reported that transferrin is regulated by estrogen hormone [34], and tamoxifen was shown to be ineffective in ER-positive breast cancer with transferrin overexpression which coincides with the tamoxifen resistance observed in HER2-positive hormone-receptor-positive breast cancer. Taken together, the ineffectiveness of tamoxifen in women with HER2-positive and hormone-receptor-positive cancer may be related to the prevalent expression of transferrin in these tumors. 

Thymosin *β*4 and *β*10 are members of a family of highly conserved small acid peptides that control the growth and differentiation of many cell types. They act as major actin-sequestering factor and play a role in cancer cell motility, invasion and metastasis [35, 36]. Thymosin *β*4 stimulates tumor metastasis by activating cell migration and angiogenesis in lung cancer and is associated with poor prognosis [37–39]. Elevations of thymosin *β*4 and *β*10 have also been reported in a number of other cancers including melanoma and breast cancer [40]. In the same tumor, the level of thymosin *β*10 was significantly higher in the cancer cells than in the normal breast parenchymal cells of the uninvolved area [41]. Its association with high-grade and poorly differentiated cancer cells is consistent with our findings of thymosin *β*10 overexpression in HER2-positive breast cancer. Further studies are required to confirm its overexpression and to determine its role in HER2-positive breast cancer.

In this study, we also reported the MS-identified protein signature predicting drug-induced tumor response in HER2-positive tumors. We found that enolase 1, vimentin, L-plastin, and ApoD predicted a favorable response of HER2-positive tumors. In contrast, elevated peroxiredoxin 5 and heat shock proteins 70 were found in nonresponding HER2-positive tumors. 

Enolase 1(ENO1), a phosphopyruvate dehydratase, is a key glycolytic enzyme involved in anaerobic metabolism under hypoxic conditions of cancer growth, and a cell surface plasminogen receptor for tumor invasion. Overexpression of ENO1 in breast, and lung cancers is associated with tumor progression and rapid tumor growth [42]. While our study did not specifically studying the prognostic value of ENO1, the observation of ENO1 in HER2-positive tumors supports the prognostic importance of this molecule. While it was seen in the more aggressive subtype of breast cancer, our study showed ENO1 elevation in HER2-positive tumors seemed to indicate a better tumor response to chemotherapy. 

Vimentin is a member of the intermediate filament family. Along with microtubules and actin microfilaments, vimentin is an integral component of the cell cytoskeleton. In cancer, altered vimentin level is associated with a dedifferentiated phenotype, increased motility, invasiveness, and poor clinical prognosis [43, 44]. Vimentin overexpression was found in 90.5% of grade III breast carcinomas [45] which may explain its presence in both HER2-positive breast cancer and in TNBC. Our study found that vimentin, although an aggressive marker for breast cancer growth, is another indicator for a favorable tumor response to chemotherapy. L-plastin is an actin-binding protein involved in cancer cell migration, invasion, and metastasis, and its expression in breast cancer cell lines correlates with the degree of invasiveness [30, 46]. In this paper, L-plastin overexpression in HER2-positive breast cancer was associated with a likelihood of pCR. 

In contrast to those molecules associated with favorable tumor response to neoadjuvant therapy, high levels of Prx V in HER2-positive breast cancers were found to be associated with poor response to the same chemotherapy regimen. Peroxiredoxins (Prxs) represent a novel group of peroxidases containing high antioxidant activity involved in cell differentiation and apoptosis [47], and Prx V is particularly effective in reducing reactive oxygen species (ROS). Moreover, Prx V is found in peroxisomes and mitochondria where protection against ROS is mostly needed. The antioxidant activity of Prx V may be associated with drug resistance of the tumor cells. 

While some molecules are unique to the characteristics of individual subtype of breast cancer, Hsp70 overexpression was found by us to be associated with drug resistance in both HER2-positive and TNBC tumors. Heat shock proteins are overexpressed in a wide range of human cancers and are implicated in tumor cell proliferation, differentiation, death, invasion, metastasis, and immune recognition [48]. Consistent with the cellular functions of Hsp70, clinically it has been correlated with poor prognosis in breast, endometrial, cervical, and bladder cancers. Others have also reported that Hsp70 mediated drug resistance through its inhibitory effect on chemotherapy-induced tumor cell apoptosis [48–50]. 

In TNBC tumors, a list of different proteins was found to be overexpressed in tumors resistant to neoadjuvant chemotherapy. In addition to Hsp70, proteins such as periostin precursor (OSF-2), RhoA, actinin *α*4, cathepsin D preproprotein, and annexin 1 predicted a poor response of TNBC to treatment. Although all of them were known to have important cancer biological properties, they have not been linked to chemotherapy susceptibility until now. 

Periostin was originally identified in a mouse osteoblastic cell line as an extracellular matrix adhesion protein for pre-osteoblasts. In addition to forming bones, teeth, and heart, periostin was recently found to be overexpressed in various types of human cancer. Periostin interacts with multiple cell-surface receptors (most notable integrins) and signals via the PI3-K/Akt and other pathways to promote cancer cell survival, epithelial-mesenchymal transition, invasion, and metastasis [51]. In breast cancer, periostin was found upregulated at both the mRNA and protein levels [51–55]. Activation of the Akt/PKB cellular survival pathway with consequential protection of tumor cells and endothelial cells from stress-induced cell death [51, 56] may contribute to the periostin-mediated drug resistance in cancer. To our knowledge, this is the first paper to link periostin to drug resistance in TNBC. 

RhoA is a member of the Ras superfamily. It is involved in the regulation and timing of cell division. It is a small GTPase protein known to regulate the actin cytoskeleton in the formation of stress fibers. RhoA protein levels were significantly increased in breast cancer compared with the matched normal tissue. It has been reported by Fritz et al. that an elevated RhoA protein level correlated with increasing breast tumor grade and poor prognosis [57]. 

Actinin *α*4 is another interesting protein that we found to indicate a poor tumor response to neoadjuvant therapy. It is thought that the actinin *α*4 cross-links actin filaments and connects the actin cytoskeleton to the cell membrane. The accumulation of actinin *α*4 in the cytoplasm is related to tumor invasiveness and metastasis, probably by enhancing cell motility, and was suggested to be a novel prognosticator in patients with ovarian and breast cancer [58]. 

Cathepsin D, an acid protease, is active in intracellular protein breakdown and is involved in the pathogenesis of several diseases. Its preproprotein secreted by cancer cells, acting as a mitogen on both cancer and stromal cells, stimulates both proinvasive and prometastatic properties of cancer cells. Many studies found that cathepsin D preproprotein/cathepsin D level represents an independent prognostic factor in a variety of cancers and is, therefore, considered to be a potential target for anticancer therapy [59]. Others have also shown that overexpression of cathepsin D in human breast cancers is associated with a higher risk of relapse and metastasis [59–61]. In our study, cathepsin D preproprotein appeared to be a drug-resistant marker in TNBC. 

Although many proteins identified in this pilot study are interesting with promising potential, this study has several limitations. First, the tumors used in this study were collected from a clinical trial which provided many controlled clinical data; however, the sample size available for proteomic analysis was small. As a result, the findings derived from a small sample size always warrant a cautious interpretation. Second, the HER2-positive group consisted of tumors with different ER and PR status which might interfere with the conclusion. The potential false associations with HER2 might be solved by stratifying the HER2-positive tumors according to hormonal receptor status in a larger study. Lastly, the HER2-positive patients in this study were randomized to receive either chemotherapy alone or chemotherapy with Herceptin. The selected drug-resistant markers may represent the resistance not only to the chemotherapy but also to Herceptin. 

In summary, our study has led to the identification of a list of important breast cancer proteins. The study also suggests that MS-based protein profiling may be an important tool in discovery of cancer biosignatures for tumor subtyping and prediction of treatment outcome. When sufficiently validated, some of these candidate protein markers could be used to improve breast cancer care. In addition, due to the heterogeneous and complex nature of the breast cancer tissue specimens, more refined methods need to be developed to maximize the protein identification to allow the capture of the best protein candidate markers for clinical use. 

## Supplementary Material

The error rates of tumor classification predicted by different sets of proteins in each model (SVM, KNN, DLDA, PAM, and SOM) were estimated by leaving-one-out test (GEPAS, version 4.0, http://www.gepas.org).
File A: SVM had the lowest error rate (10%, 4/39) in tumor classification using 20 proteins listed in Table 3.
File B and C: KNN had the lowest error rate (9%, 1/11) in predicting HER2-positive tumor response using 20 proteins listed in Table 4. By using KNN = 1 method, 100% (4/4) tumors in NR and 85.7% (6/7) tumors in pCR were correctly grouped.
File D and E: DLDA had the lowest error rate (18%, 2/11) in predicting TNBC tumor response using 30 proteins listed in Table 5. 85.7% (6/7) tumors in the R group and 75% (3/4) tumors in IR/NR group were correctly classified.

## Figures and Tables

**Table:** (a) Clinical characteristics of 11 TNBC tumors

LTQ Orbitrap sample ID	Patient age	Ethnicity	TR %	Response	T stage	Histological type	ER	PR	FISH R/G ratio	Neoadjuvant
#1	61	White	80	R	T3	IDC	−	−	1.10	TC
#2	29	Hispanic	−60	NR	T3	IDC	−	−	0.92	TC
#5	55	Hispanic	100	R (pCR)	T3	IDC	−	−	0.92	TC
#6	54	Hispanic	100	R (pCR)	T3	IDC	−	−	1.01	TC
#7	40	Asian	45	IR	T3	IDC	−	−	1.17	TC
#8	44	White	100^a^	R (pCR)	T3	IDC	−	−	1.00	TC
#9	49	Hispanic	48	IR	T4	IDC	−	−	1.10	TC
#10	53	White	100	R (pCR)	T3	IDC	−	−	1.20	TC
#11	84	Asian	100	R (pCR)	T4	IDC	−	−	1.03	TC
#36	45	Hispanic	30	IR	T3	IDC	−	−	1.27	TC
#37	38	White	100	R (pCR)	T2	IDC	−	−	1.10	TC

^a^LN positive without residual primary cancer.

**Table:** (b) Clinical characteristics of 28 HER2+ tumors

LTQ Orbitrap sample ID	Patient age	Ethnicity	TRR %	Response	T stage	Histological type	ER	PR	FISH R/G ratio	Neoadjuvant
#17	38	White	40	IR	T3	IDC	+	−	12.4	TC
#18	63	Asian	100	R (pCR)	T3	IDC	+	−	12.7	TCH
#19	57	White	100	R (pCR)	T3	IDC	−	−	4.6	TC
#20	56	Asian	78.2	R	T4	IDC	+	−	10.71	TC
#21	51	Black	56	IR	T3	IDC	−	−	19.97	TCH
#22	31	White	45.5	IR	T3	IDC	+	+	2.2	TC
#23	55	White	80	R	T4	IDC	+	+	3.8	TC
#24	45	Asian	75	IR	T4	IDC	+	+	2.7	TCH
#25	42	Hispanic	63.5	IR	T4	IDC	+	−	2.5	TC
#26	50	White	67.1	IR	T3	IDC	−	−	2.41	TCH
#27	33	White	82.9	R	T3	IDC	+	+	3.03	TCH
#28	40	White	66.7	IR	T3	IDC	−	−	8.1	TC
#29	35	Hispanic	100^a^	R (pCR)	T3	IDC	−	−	42.2	TCH
#30	44	White	97.3	R	T4	IDC	−	−	4.2	TC
#31	30	White	−7.7	NR	T3	IDC	+	−	5	TC
#32	57	White	25%	NR	T4	IDC	+	−	>4	TCH
#33	37	White	33.3	IR	T2	IDC	+	+	9.49	TC
#34	36	Black	25	NR	T3	IDC	−	−	5.1	TC
#35	42	White	60	IR	T2	IDC	+	+	4.5	TCH
#38	55	White	42.3	IR	T4	IDC	−	−	3.9	TCH
#39	47	White	100^b^	R (pCR)	T3	IDC	+	+	>20	TCH
#40	50	Asian	100^b^	R (pCR)	T3	IDC	+	+	4.19	TCH
#41	58	White	50	IR	T4	IDC	+	+	2.1	TCH
#42	40	Asian	60	IR	T2	IDC	−	−	16	TC
#43	37	White	−85.6	NR	T3	IDC	+	+	3.1	TC
#44	49	White	100^b^	R (pCR)	T2	IDC	+	−	7.7	TCH
#45	55	Asian	92.6	R	T3	IDC	−	−	9.9	TC
#46	41	Hispanic	100	R (pCR)	T2	IDC	−	−	9.2	TC

^a^LN positive and residual DCIS; ^b^residual DCIS only.

**Table 2:** The 20 most abundant proteins shared by both HER2-positive and TNBC tumors.

Identified proteins	Accession no.	MW
Apolipoprotein A-I	gi∣90108664	28 kDa
Vimentin	gi∣62414289	54 kDa
Enolase 1	gi∣4503571	47 kDa
Alpha-1 antitrypsin	gi∣157086955	45 kDa
Triosephosphate isomerase 1	gi∣4507645 (+2)	27 kDa
Cyclophilin A	gi∣1633054	18 kDa
Apolipoprotein D	gi∣619383	28 kDa
Cofilin 1	gi∣5031635	19 kDa
Chaperonin	gi∣31542947	61 kDa
Transgelin 2	gi∣4507357	22 kDa
Heat shock 70 kDa protein 5	gi∣16507237	72 kDa
Tumor rejection antigen (gp96) 1	gi∣4507677	92 kDa
S100 calcium-binding protein A11	gi∣5032057	12 kDa
Lumican precursor	gi∣4505047	38 kDa
Tropomyosin 4	gi∣4507651	29 kDa
ATP synthase, H+ transporting, mitochondrial F1 complex	gi∣32189394	57 kDa
Prosaposin isoform a preproprotein	gi∣11386147	58 kDa
Profilin	gi∣157838211 (+4)	15 kDa
Heat shock 70 kDa protein 8 isoform 1	gi∣5729877	71 kDa
Annexin 5	gi∣4502107	36 kDa

**Table 3:** Top 20 differentially expressed proteins selected by supervised classification methods for classifying two tumor subtypes.

Rank	Accession no.	Protein name	MW	HER2+/TNBC mean	Subcellular location	Function
1	gi∣10946578	Thymosin *β*4	5 kDa	2.99	Cytoplasm, cytoskeleton	For cytoskeletal binding, involved in cell growth and maintenance
2	gi∣4507521	Transketolase	68 kDa	4.20	Cytosol	Involved in metabolism. Associated with cell proliferation of uterine and cervical cancer.
3	gi∣1633054	Cyclophilin A	18 kDa	2.45	Cytoplasma	Involved in accelerate the folding of proteins
4	gi∣73858568	Complement component 1 inhibitor	55 kDa	0.33	Secreted	Regulating the complement cascade
5	gi∣4557871	Transferrin	77 kDa	16.38	Secreted	Essential for cell growth and iron-dependent metabolic processes
6	gi∣90111766	Keratin type I cytoskeletal 19	44 kDa	11.29	Cytoskeleton	Involved in metastatic progression of breast cancer
7	gi∣10863895	Thymosin *β*10	5 kDa	2.25	Cytoplasm, cytoskeleton	For cytoskeletal binding, involved in cell growth and maintenance
8	gi∣5031863	Galectin-3-binding protein	65 kDa	0.41	Secreted	Modulating cell-cell and cell-matrix interactions
9	gi∣4505753 (+1)	Phosphoglycerate mutase 1	29 kDa	2.51	Cytosol	Involved in glycolysis
10	gi∣5174391	Aldo-keto reductase family 1, member A1	37 kDa	0.30	Cytosol	Involved in the reduction of biogenic and xenobiotic aldehydes
11	gi∣21361176	Aldehyde dehydrogenase 1A1	55 kDa	0.39	Cytoplasm	Detoxifying enzyme responsible for oxidating of intracellular aldehydes. A marker for cancer stem cells
12	gi∣4505185	Macrophage migration inhibitory factor	12 kDa	0.36	Secreted, cytoplasm	Involved in integrin signaling pathways
13	gi∣4507645 (+2)	Triosephosphate isomerase 1	27 kDa	2.49	Cytosol, nucleus	Fatty acid biosynthesis, gluconeogenesis, glycolysis, lipid synthesis
14	gi∣4930167	Aldolase A	39 kDa	6.41	Extracellular, cytoskeleton	Involved in glycolysis
15	gi∣116241280	Adenylyl cyclase-associated protein 1 (CAP 1)	52 kDa	3.03	Membrane	Regulating filament dynamics, cell polarity and signal transduction,
16	gi∣21624607 (+5)	Coactosin-like 1	16 kDa	0.42	Cytoplasm, cytoskeleton	Regulating the actin cytoskeleton
17	gi∣160420317	Filamin A, alpha isoform 2	281 kDa	3.10	Cytoplasm	Anchoring transmembrane proteins to the actin cytoskeleton, scaffold for cytoplasmic signaling proteins
18	gi∣6005942	Valosin-containing protein	89 kDa	3.26	Cytosol, nucleus	Fragmentation of Golgi stacks during mitosis and reassembly
19	gi∣5174539	Cytosolic malate dehydrogenase	36 kDa	2.52	Cytoplasm	Involved glycolysis, oxidation reduction, and tricarboxylic acid cycle
20	gi∣33286418 (+2)	Pyruvate kinase 3	58 kDa	6	Cytoplasm, nucleus	Involved in glycolysis

**Table 4:** Top 20 proteins predicting tumor response to neoadjuvant treatment in HER2-positive tumors.

Rank	Protein name	Accession no.	pCR/NR mean	Subcellular location	Function
1	Enolase 1	gi∣4503571	2.59	Cytoplasm, cell membrane	Multifunctional enzyme
2	Heterogeneous nuclear ribonucleoprotein A2/B1 isoform B1	gi∣14043072	3.51	Nucleus, cytoplasm	Pre-mRNA processing
3	Heat shock 70 kDa protein 1	gi∣75061728	0.24	Cytoplasm	Stress response
4	Vimentin	gi∣62414289	9.94	Cytosol	Class III intermediate filaments
5	Vesicle amine transport protein 1	gi∣18379349	0.50	Cytoplasmic vesicle membrane	Neurotransmitter transport
6	Coronin, actin-binding protein, 1A	gi∣5902134	2.00	Cytoplasm	Component of the cytoskeleton of highly motile cells
7	Fatty acid-binding protein 4	gi∣4557579 (+1)	0.23	Cytoplasm, nucleus	Lipid transport protein
8	Peroxiredoxin 5	gi∣15826629	0.37	Mitochondrion, cytoplasm, peroxisome	Antioxidant, oxidoreductase peroxidase
9	Heat shock 70 kDa protein 9	gi∣24234688	0.15	Mitochondrion	Control of cell proliferation and cellular aging
10	Leucine aminopeptidase 3	gi∣41393561	2.94	Cell membrane, secreted	Cell-cell signaling
11	Apolipoprotein D	gi∣619383	2.90	Secreted	Lipid metabolic process
12	L-plastin	gi∣4504965	3.14	Cytoplasm, cell membrane	Activation of T cells, intracellular protein transport
13	Anterior gradient protein 2 homolog precursor	gi∣5453541	0.11	Secreted, endoplasmic reticulum	Mucus secretion
14	Heat shock 10 kDa protein 1	gi∣4504523	0.37	Mitochondrion	Stress response
15	ATP synthase, H+ transporting, mitochondrial F1 complex	gi∣4757810	0.41	Mitochondrion	Proton-transporting ATP synthase complex assembly
16	Glutathione transferase	gi∣20664358 (+5)	3.29	Cytoplasm	Glutathione metabolic process
17	Chaperonin	gi∣31542947	0.33	Mitochondrion	Stress response
18	Complement component 3 precursor	gi∣115298678	3.00	Secreted	Activation of the complement system
19	Heterogeneous nuclear ribonucleoprotein D isoform a	gi∣14110420	2.19	Nucleus, cytoplasm	Transcription regulation
20	Malate dehydrogenase	gi∣6648067 (+1)	0.22	Cytoplasm	Tricarboxylic acid cycle

**Table 5:** Top 30 proteins predicting tumor response to neoadjuvant chemotherapy in TNBC tumors.

Rank	Protein name	Accession no.	R/IR + NR mean	Subcellular location	Function
1	Heat shock 70 kDa protein 8 isoform 1	gi∣5729877	0.32		Stress response
2	Periostin precursor (PN) (osteoblast-specific factor 2)	gi∣93138709	0.31	Nucleus	Transcription regulation
3	Cyclophilin A	gi∣1633054	0.41	Secreted	Cell attachment adhesion and spreading
4	Tyrosine 3/tryptophan 5-monooxygenase activation protein	gi∣5803225 (+1)	3.71	Nucleus	Protein binding
5	Profilin	gi∣157838211 (+4)	0.32	Cytoplasm, cytoskeleton	Actin cytoskeleton organization
6	Cardiac muscle alpha actin 1 proprotein	gi∣4885049	0.08	Cytoplasm, cytokeleton	actin filament-based movement, apoptosis
7	Beta actin	gi∣4501885	0.22	Cytoplasm, cytokeleton	Cell motility
8	Caldesmon (CDM)	gi∣2498204	0.42	Cytoplasm, cytokeleton	Actin- and myosin-binding protein
9	Tubulin *β*5	gi∣7106439	0.19	Cytosol	Major constituent of microtubules
10	Tropomyosin 2 (beta) isoform 1	gi∣42476296	0.11	Cytoplasm, cytokeleton	Binding to actin filaments
11	Actinin, *α*4	gi∣12025678	0.11	Nucleus, cytoplasm	Protein transport
12	Ras homolog gene family, member A (RhoA)	gi∣10835049 (+4)	0.33	Cytoplasm, cell membrane	Regulating a signal transduction pathway
13	Heterogeneous nuclear ribonucleoprotein K	gi∣13384620	0.33	Cytoplasm, nucleus	Pre-mRNA-binding proteins
14	Tubulin *α*1	gi∣6755901	0.36	Cytosol	Major constituent of microtubules
15	Tropomyosin 4	gi∣4507651	0.35	Cytoplasm, cytokeleton	Binds to actin filaments
16	Complement component 1 inhibitor precursor	gi∣73858568	4.57	Secreted	Complement pathway
17	ATP synthase, H+ transporting, mitochondrial F1 complex	gi∣4757810	0.43	Mitochondrion	Proton-transporting ATP synthase complex assembly
18	Calnexin precursor	gi∣10716563	0.42	Endoplasmic reticulum membrane, cell membrane	Calcium-binding protein
19	Eukaryotic translation elongation factor 1 alpha 1	gi∣4503471	0.29	Cytoplasm	Protein biosynthesis
20	Annexin I	gi∣4502101	0.25	Nucleus, cytoplasm, membrane	Calcium/phospholipid-binding protein
21	Triosephosphate isomerase 1	gi∣4507645 (+2)	0.35	Cytosol, nucleus	Fatty acid biosynthesis, gluconeogenesis, glycolysis, lipid synthesis
22	Cathepsin D preproprotein	gi∣4503143	0.35	Lysosome	proteolysis
23	Alpha glucosidase II alpha subunit isoform 2	gi∣38202257	0.19	Cytosol	Glycan metabolism, N-glycan metabolism
24	Tyrosine 3-monooxygenase/tryptophan 5-monooxygenase activation protein	gi∣4507949 (+1)	0.42	Nucleus	Protein binding
25	Thymosin *β*10	gi∣10863895	0.42	Cytoplasm, cytokeleton	cytoskeleton organization
26	Aconitase 2 precursor	gi∣4501867	0.44	Mitochondrion	Carbohydrate metabolism, tricarboxylic acid cycle
27	Heterogeneous nuclear ribonucleoprotein D isoform a	gi∣14110420	0.48	Nucleus, cytoplasm	Transcription regulation
28	Serine (or cysteine) proteinase inhibitor	gi∣32454741	0.25	Secreted	Inhibits activated protein C, plasminogen activator
29	Lumican precursor	gi∣4505047	0.28	Secreted	Binds to laminin
30	Apolipoprotein D	gi∣619383	2.86	Secreted	Transport

## Abbreviations

DLDA: Diagonal linear discriminant analysis
G3BP: Galectin-3-binding protein
GEPAS: Gene expression pattern analysis suite
IR: Intermediate responders (>25% but ≤75% tumor regression)
KNN: K nearest neighbor
MS: Mass spectrometry
NR: Nonresponders, chemoresistant tumors (≤25% tumor regression)
PAM: Prediction analysis with microarrays
pCR: Pathological complete response, no residual cancer found at primary tumor site
R: Responders (>75% of tumor regression)
SOM: Self-organizing map
SVM: Support vector machines
TC ± H: Taxotere/Carboplatin/±Herceptin treatment
TNBC: Triple negative breast tumors
TRR: Tumor regression rate.
