# Supplementary material for: Proteomic-Based Biosignatures in Breast Cancer Classification and Prediction of Therapeutic Response
Source: Int J Proteomics. 2011 Oct 24;2011:896476. doi: 10.1155/2011/896476 (PMC3202144; doi:10.1155/2011/896476)
Supplement: Supplementary file 1 — The error rates of tumor classification predicted by different sets of proteins in each model (SVM, KNN, DLDA, PAM, and SOM) were estimated by leaving-one-out test (GEPAS, version 4.0, http://www.gepas.org). File A: SVM had the lowest error rate (10%, 4/39) in tumor classification using 20 proteins listed in Table 3. File B and C: KNN had the lowest error rate (9%, 1/11) in predicting HER2-positive tumor response using 20 proteins listed in Table 4. By using KNN = 1 method, 100% (4/4) tumors in NR and 85.7% (6/7) tumors in pCR were correctly grouped. File D and E: DLDA had the lowest error rate (18%, 2/11) in predicting TNBC tumor response using 30 proteins listed in Table 5. 85.7% (6/7) tumors in the R group and 75% (3/4) tumors in IR/NR group were correctly classified. [file 896476.f1.doc]

**Additional files**

**A.** Error rate vs*.* number of proteins selected for assigning tumor subtype by different classification methods


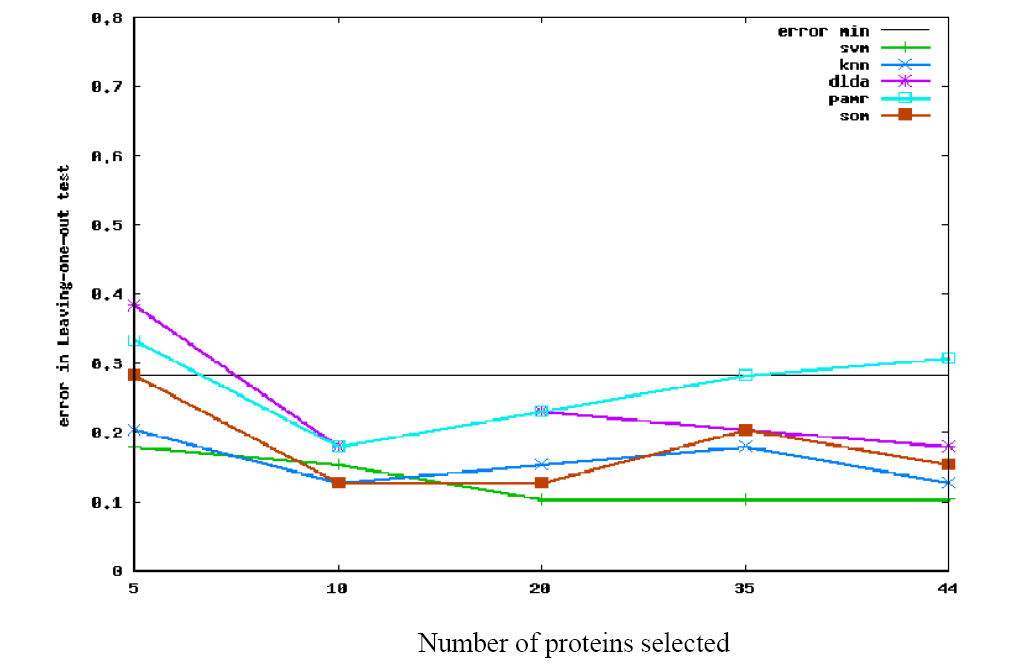


B. Error rate vs*.* number of proteins selected to predict tumor response to neoadjuvant treatment in HER2 positive tumors by different classification methods


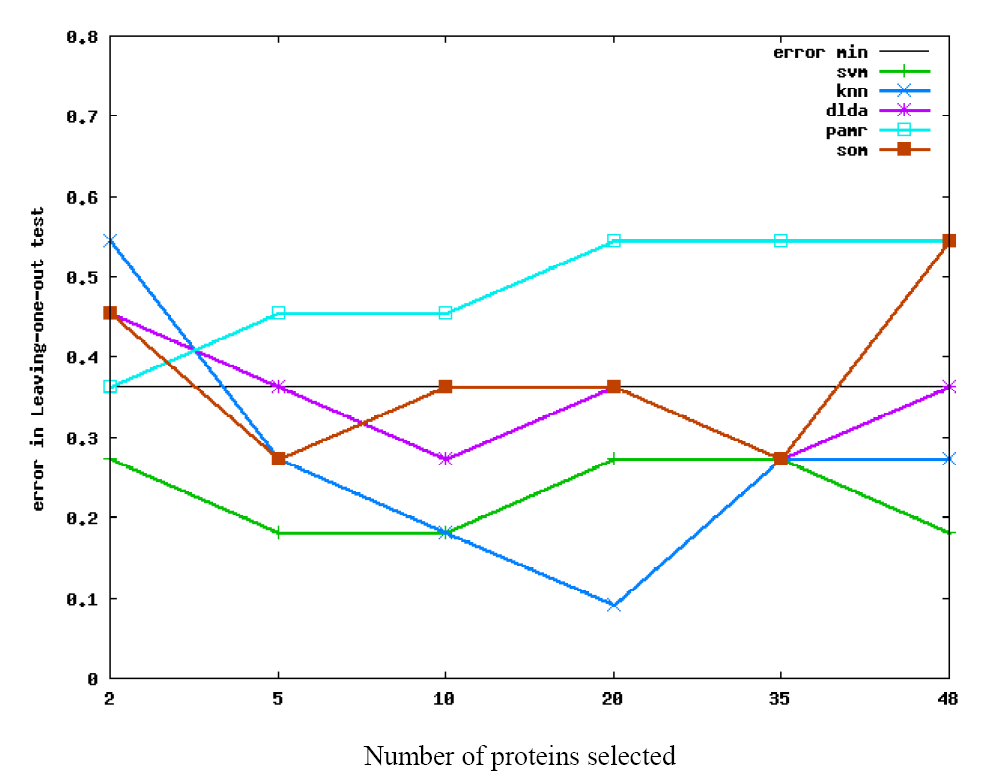


C. Different set of proteins predicting HER2+ tumors response selected by KNN method (KNN=1)

| Array | **Observed tumor response** | **5 proteins prediction** | **10 proteins prediction** | **20 proteins prediction** | **35 proteins prediction** | **48 proteins prediction** |
| --- | --- | --- | --- | --- | --- | --- |
| **HER2positive_NR#31** | NR | pCR | NR | NR | NR | NR |
| **HER2positive_NR#32** | NR | NR | NR | NR | pCR | pCR |
| **HER2positive_NR#34** | NR | pCR | pCR | NR | pCR | pCR |
| **HER2positive_NR#43** | NR | NR | NR | NR | NR | NR |
| **HER2positive_pCR#18** | pCR | pCR | pCR | pCR | pCR | pCR |
| **HER2positive_pCR#19** | pCR | pCR | pCR | pCR | pCR | pCR |
| **HER2positive_pCR#29** | pCR | pCR | pCR | pCR | pCR | pCR |
| **HER2positive_pCR#39** | pCR | NR | NR | NR | NR | NR |
| **HER2positive_pCR#40** | pCR | pCR | pCR | pCR | pCR | pCR |
| **HER2positive_pCR#44** | pCR | NR | pCR | pCR | pCR | pCR |
| **HER2positive_pCR#46** | pCR | pCR | pCR | pCR | pCR | pCR |

D. Error rate vs. number of proteins selected to predict TNBC tumor response to neoadjuvant treatment by different classification methods

E. Different set of proteins predicting TNBC tumors response selected by DLDA method

| **Array** | **Observed tumor response** | **5 proteins prediction** | **10 proteins prediction** | **20 proteins prediction** | **30 proteins prediction** | **50 proteins prediction** | **63 proteins prediction** |
| --- | --- | --- | --- | --- | --- | --- | --- |
| **TNBC_IR#36** | IR/NR | R | R | IR/NR | IR/NR | IR/NR | IR/NR |
| **TNBC_IR#7** | IR/NR | R | R | R | IR/NR | IR/NR | IR/NR |
| **TNBC_IR#9** | IR/NR | R | R | R | R | R | R |
| **TNBC_NR#2** | IR/NR | IR/NR | IR/NR | IR/NR | IR/NR | IR/NR | IR/NR |
| **TNBC_R#1** | R | R | R | R | R | R | R |
| **TNBC_pCR#10** | R | R | R | R | R | R | R |
| **TNBC_pCR#11** | R | R | R | R | R | R | R |
| **TNBC_pCR#37** | R | R | R | R | R | R | R |
| **TNBC_pCR#5** | R | R | R | R | R | R | R |
| **TNBC_pCR#6** | R | R | R | R | R | R | R |
| **TNBC_pCR#8** | R | IR/NR | IR/NR | IR/NR | IR/NR | IR/NR | IR/NR |
